# Expression levels of thymosin α1 in acute myocardial infarction patients and its correlation to cardiac function

**DOI:** 10.3389/fcvm.2025.1635557

**Published:** 2025-09-12

**Authors:** Liang Liu, Zhen-Fa Zhou, Xian Jin, Yu Chen, Cui-Fen Hu, Cheng-Xing Shen

**Affiliations:** ^1^ Department of Cardiology, Shanghai Sixth People's Hospital Affiliated to Shanghai Jiao Tong University School of Medicine, Shanghai, China; ^2^ Department of Cardiovascular Medicine, Fuzhou First Hospital Affiliated with Fujian Medical University, Fuzhou, China; ^3^ State Key Laboratory of Molecular Engineering of Polymers, Fudan University, Shanghai, China; ^4^ Department of Ultrasound in Medicine, Minhang Hospital, Fudan University, Shanghai, China

**Keywords:** thymosin α1, cardiac function, acute myocardial infarction, biomarker, therapy

## Abstract

**Background:**

Early prediction of heart failure (HF) after acute myocardial infarction (AMI) remains a clinical challenge. There is a lack of studies investigating Thymosin α1 expression levels in AMI patients and its relationship with cardiac function post-AMI.

**Methods:**

This retrospective analysis included patients with AMI from December 2019 to February 2022. The baseline data of two groups were collected. Thymosin α1 expression level of peripheral blood plasma in AMI patients was examined by ELISA. Logistic regression analysis was applied to evaluate risk factors in-hospital cardiac dysfunction after emergency PCI in AMI patients. Receiver operating characteristic (ROC) curve was used to analyze the predictive value of the biomarker.

**Results:**

A total of 307 hospitalized patients were enrolled in this study, divided into AMI group (*n* = 274) and non-AMI group (*n* = 33). The expression level of thymosin α1 in the AMI group was significantly higher than in the non-AMI group. The AMI patients were divided into two subgroups based on the EF values. The sample size was 64 (EF < 50%) and 210 (EF ≥ 50%), respectively. The expression of thymosin α1 in the EF ≥ 50% group was significantly higher than EF < 50% group. Spearman's correlation analysis demonstrated that thymosin α1 was positively correlated with the EF value. Logistic multivariate analysis suggested that thymosin α1, NT-proBNP, and creatine kinase were independent predictors of cardiac function after AMI. The AUC of thymosin α1, NT-proBNP, and creatine kinase was 0.614, 0.714, and 0.724, respectively.

**Conclusion:**

Thymosin α1 may serve as a potential biomarker to predict cardiac function following AMI. This study may provide novel insights into the potential therapeutic targets for HF following AMI.

## 1 Introduction

Coronary heart disease remains one of the leading causes of morbidity and mortality worldwide (1). Acute myocardial infarction (AMI) is the most serious manifestation of coronary heart disease, which seriously threatens human health and increases social burden (2). With the advancement of contemporary medicine, especially the implementation of coronary intervention surgeries, many AMI patients can have their culprit vessels opened in a timely and effective manner (3). As a result, the mortality rate has significantly decreased compared to the past (3). However, the complications of AMI cannot be ignored, as they seriously affect the patient's life and health as well as their quality of life. AMI is frequently associated with numerous complications, among which new-onset or chronic heart failure (HF) after discharge is a prevalent one (4). The emergence of HF after an MI can notably enhance the mortality rate and the risk of recurrent hospitalizations for this patient group (5). Therefore, the early and accurate detection of post-MI cardiac dysfunction through clinically effective laboratory indicators, and the subsequent administration of effective treatment, constitute the most efficacious strategies to mitigate the long-term adverse prognosis of this patient group.

Thymosins were initially purified from the calf thymus by Allan L. Goldstein in 1966 as a biologically active substance (6). Subsequent research has disclosed that numerous tissues in the human body can secrete thymosin (7). Due to the isoelectric point, thymosins are divided into isoforms α (pI < 5), β (5 < pI < 7), and γ (pI > 7) (7). T-*β*4 is the most abundant *β*-thymosin (constituting approximately 70%–80%) and holds numerous biological effects, including cell proliferation and apoptosis, inflammatory responses, and fibrosis within the body (7–9). A significant number of studies have been conducted on the role of T-*β*4 in myocardial fibrosis and cardiac function after AMI, suggesting its potential protective mechanisms in the myocardium following AMI (10, 11). Thymosin α1 is related to various biological effects in the body and mainly participates in immune regulation (12). It can promote the maturation and differentiation of T lymphocytes, stimulate the secretion of various lymphokines by mature T cells and NK cells, and augment the body's immune response and resistance to infection (7, 12). Moreover, it has an anti-inflammation protective effect to reduce the damage of central nervous system diseases (13). Given the regulation of thymosin α1 on the immune function of the body, in clinical practice, thymosin α1 is mainly used for the combined treatment of cancers (14), the treatment of hepatitis B virus (15), and the treatment of HIV patients (16). The inflammatory response following AMI plays a crucial role in the progression of myocardial injury. However, the role of Thymosin α1 post-AMI remains poorly understood. The potential of Thymosin α1 as a biomarker for myocardial injury and cardiac dysfunction following AMI is yet to be fully explored.

This study focuses on this issue. By comparing with the control group, the expression level of Thymosin α1 in the peripheral blood of patients with AMI and its relationship with early-onset HF after AMI are clarified. The goal is to identify a novel biomarker for the early clinical detection of HF following AMI, thereby facilitating early intervention and improving long-term outcomes for this patient population.

## 2 Materials and methods

### 2.1 Patients

From December 2019 to February 2022, a total of 307 patients were consecutively enrolled as the study subjects, including 274 patients with AMI as the case group, and 33 patients without AMI at enrollment and no prior history of AMI as the control group. All the AMI patients met the diagnostic criteria of the 2023 ESC Guidelines for the management of acute coronary syndromes (17). For AMI patients with confirmed ST-segment elevation myocardial infarction (STEMI), the infarct-related artery (IRA) should be opened within 12 h. For NSTEMI patients, the IRA will be opened within 2, 12, 24, and 72 h according to risk stratification. Moreover, all AMI patients will receive optimal drug therapy according to the guideline (17). The exclusion criteria for this study were as follows: (a) A previous history of AMI; (b) A history of coronary artery bypass grafting (CABG) or percutaneous coronary intervention (PCI); (c) Severe hepatic/renal insufficiency or chronic obstructive pulmonary disease; (d) Disorders in the hematological, immune, or coagulation systems; (e) The presence of malignant tumors; (f) The existence of psychiatric disorders; (g) Incomplete clinical or laboratory data; (h) Any other circumstances that the researcher deems inappropriate to participate in this study. This study was approved by the Hospital Ethics Committee and the ethical number is 2021-KY-04(K)-(1). All the enrolled patients signed the informed consent form. The study was by the principles of the Declaration of Helsinki and was approved by the Hospital Ethics Committee.

### 2.2 Clinical or laboratory data

The baseline data of clinical records encompass age, gender, height, weight, systolic blood pressure, heart rate, etc. The past medical history of patients, including hypertension, diabetes, etc., was documented. Blood routine, biochemical markers, and coagulation indicators were all recorded as the fasting blood results in the early morning of patients. Markers of myocardial injury in AMI patients, including troponin, creatine kinase, N-terminal pro-B-type natriuretic peptide (NT-proBNP), etc., were all documented as the highest values during hospitalization. The LVEF assessment was performed within 7 days post-PCI for AMI patients and the Simpson method was used to measure LVEF.

### 2.3 Thymosin α1 examination by ELISA

Peripheral venous blood samples (5 ml) were collected from all subjects during the initial admission period prior to IRA revascularization. Subsequently, the venous blood was kept at room temperature for 30 min, and the serum was extracted after centrifugation at 1,000 g for 15 min using a centrifuge. The serum was stored at −80°C for subsequent use. The expression of serum thymosin α1 was determined using the enzyme-linked immunosorbent assay (ELISA) kit (Jing Mei Biotechnology, Jiangsu, China. No. JM-03731H1).

### 2.4 Statistical analysis

Continuous variables with a normal distribution were expressed as mean ± standard deviation (SD), and the independent sample *t*-test was employed for intergroup comparison; data with a non-normal distribution were expressed as median [interquartile range (IQR)], and the Mann–Whitney *U*-test was utilized for comparison between groups. Categorical variables were depicted as percentages, and the *χ*2 test or Fisher's exact test was applied for intergroup comparison.

All the significant variables identified in the intergroup comparison were enrolled in the logistic regression analysis, and the standard error and odd ratio (OR) were documented. The linear relationship between serum thymosin α1 and EF value post-AMI was assessed using the Spearman correlation analysis. The predictive value of Thymosin α1 for cardiac function following AMI was estimated by the receiver operating characteristic (ROC) curve. Statistical significance was considered as *P* < 0.05. All statistical analyses were performed using SPSS 26.0 statistical software.

## 3 Results

### 3.1 Comparison of baseline demographic and clinical data

A total of 307 patients were enrolled in this study, including 274 in the AMI group and 33 in the control group. Baseline data between the two groups, including gender, age, height, weight, BMI, systolic blood pressure, heart rate, smoking rate, drinking rate, prevalence of diabetes, and prevalence of hypertension, showed no statistically significant differences (Table 1).

**Table 1:** Comparison of clinical features between AMI group and control group.

Variable	AMI (*n* = 274)	Ccontrol (*n* = 33)	*P*-value
Male (n, %)	219 (79.9)	23 (69.7)	0.174
Age (Year)	65 (55–72)	66 (59–72)	0.817
Height (m)	1.70 (1.64–1.74)	1.70 (1.59–1.72)	0.220
Weight (kg)	70.0 (64.0–78.0)	67.0 (58.8–82.5)	0.250
BMI (kg/m^2^)	24.68 (22.64–26.57)	24.22 (22.40–26.94)	0.596
SBP (mmHg)	126 (110–141)	132 (122–140)	0.290
HR (bpm)	80 (72–88)	78 (69–90)	0.367
Smoking (n, %)	165 (60.2)	21 (63.6)	0.704
Drinking (n, %)	73 (26.6)	7 (21.2)	0.502
Diabetes (n, %)	58 (21.2)	6 (18.2)	0.690
Hypertension (n, %)	148 (54.0)	22(66.7)	0.167

SBP, systolic blood pressure; HR, heart rate.

### 3.2 Thymosin α1 level was elevated in AMI patients with EF ≥ 50%

**Figure 1:**
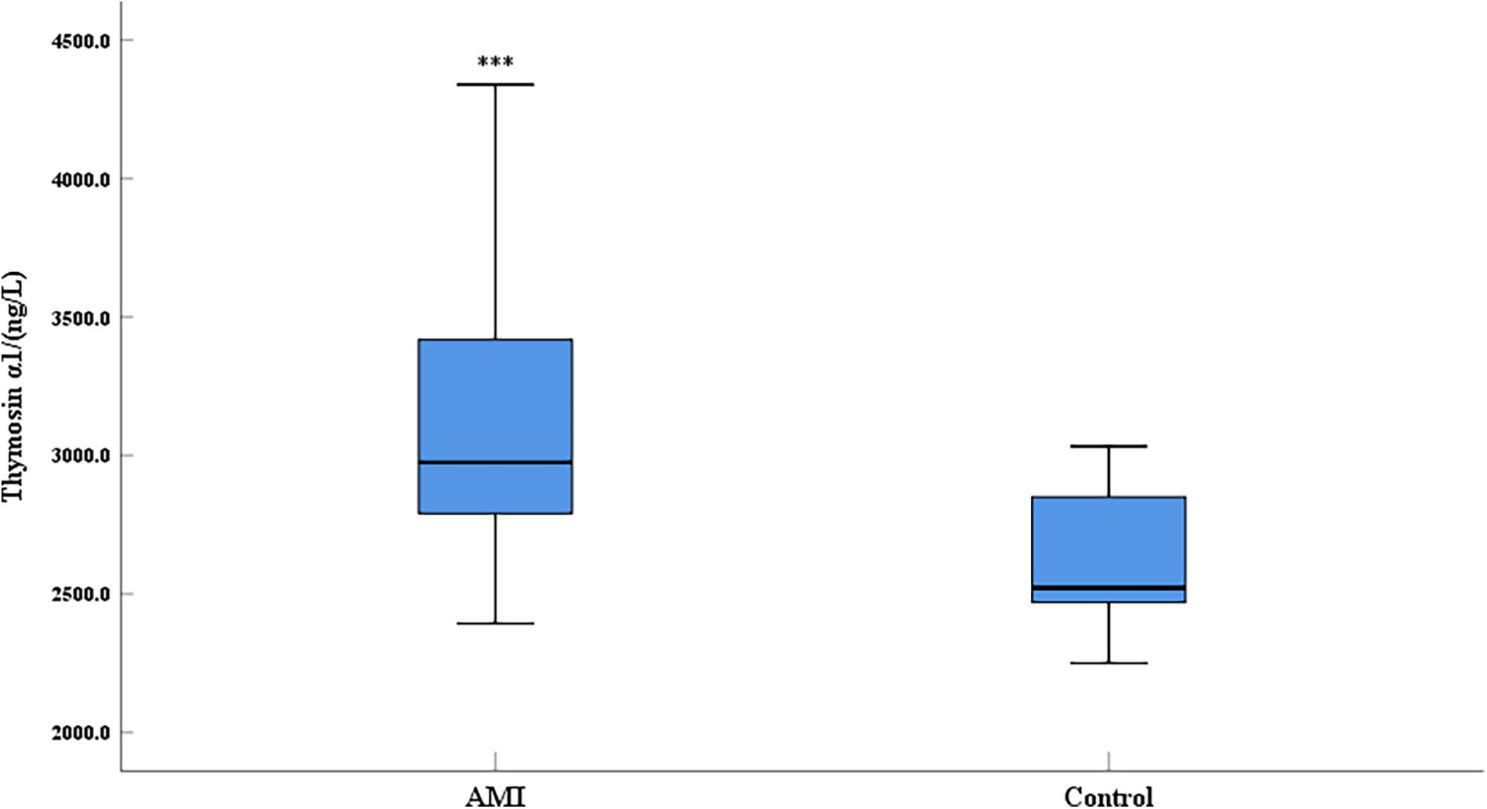
Comparison of serum thymosin α1 level between AMI group and control group (AMI: acute myocardial infarction group, control: control group; ***: *P* < 0.001).

**Figure 2:**
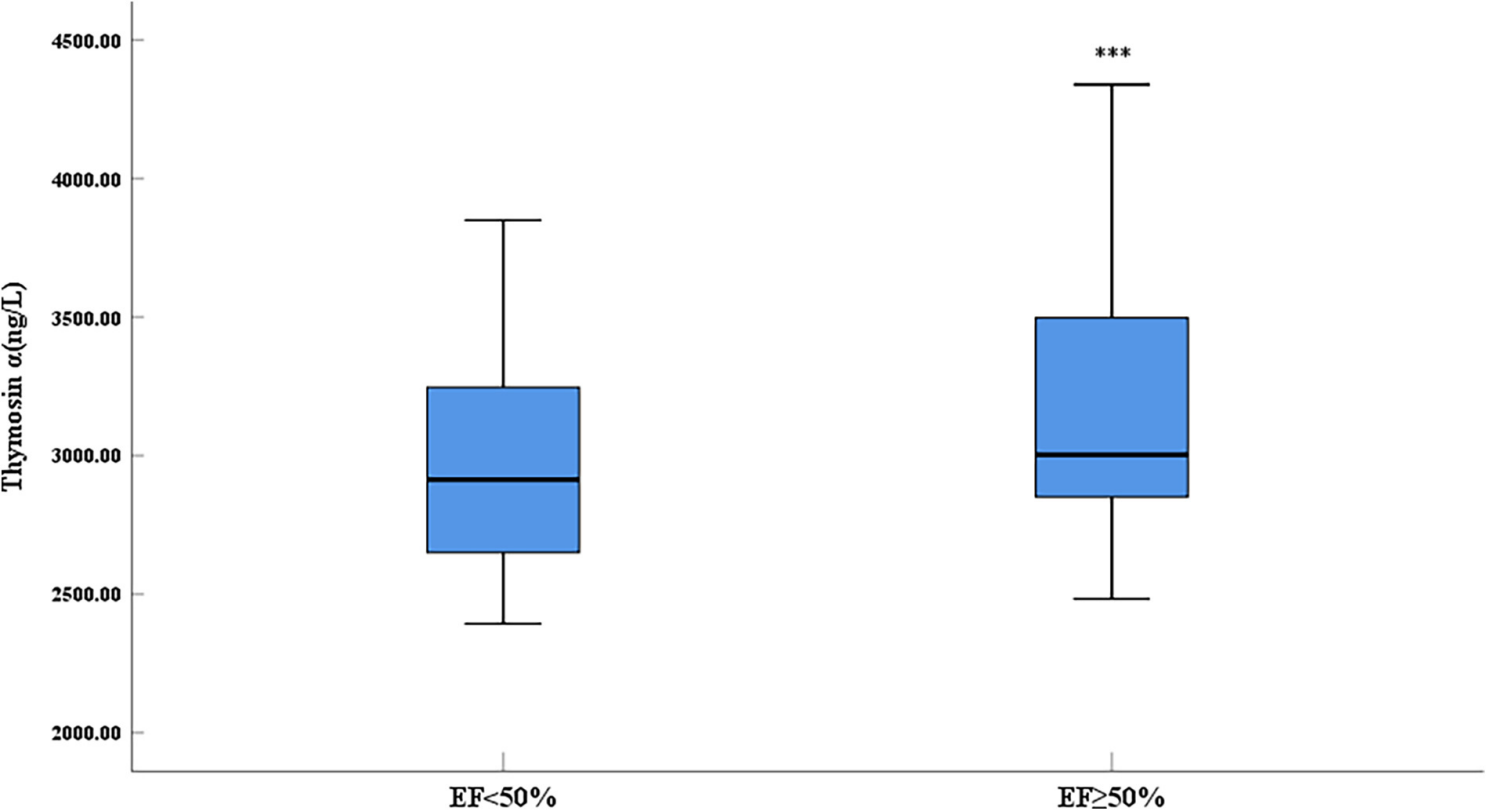
Comparison of serum thymosin α1 level between different LVEF groups in AMI patients (***: *P* < 0.001).

Compared with the control group, the expression level of thymosin α1 was significantly elevated in AMI patients (Figure 1). The 2023 Focused Update of the 2021 ESC Guidelines for the Diagnosis and Treatment of Acute and Chronic Heart Failure utilizes the EF value as a crucial basis for chronic HF stratification. HF is classified into two types: HF with reduced ejection fraction (including EF between 41% and 49%, known as HFmrEF, and EF ≤ 40%, known as HFrEF) and HF with preserved ejection fraction (EF ≥ 50%, known as HFpEF) (18). An EF of 50% is an important numerical cut-off for assessing cardiac function reduction. To further clarify the relationship between thymosin α1 and cardiac function in patients with AMI, we categorized AMI patients into two groups based on postoperative echocardiography: one group with an EF < 50% and another group with an EF ≥ 50%. Initially, according to the design, the final number of enrolled patients with an EF < 50% was 64, while the number of patients with an EF ≥ 50% was 210. Baseline data showed no statistically significant differences between the two groups in terms of gender, age, height, weight, systolic blood pressure, heart rate, smoking rate, drinking rate, prevalence of diabetes, and prevalence of hypertension, indicating that the two groups were comparable (Table 2). Furthermore, our study demonstrated that the expression levels of thymosin α1 [2,913.28 (2,648.35–3,247.05) vs. 3,002.50 (2,846.48–3,497.68), *P* = 0.006] in peripheral blood were significantly higher in patients with an EF ≥ 50% after AMI compared to those with an EF < 50% (Figure 2).

**Table 2:** Comparison of clinical features among different ejection fraction groups in AMI patients.

Variable	LVEF < 50%(*n* = 64)	LVEF ≥ 50%(*n* = 210)	*P*-value
Male (n, %)	52 (81.3)	154 (73.3)	0.199
Age (Year)	65.00 (60.00–72.75)	64.00 (54.00–72.00)	0.123
Height (m)	1.70 (1.65–1.75)	1.70 (1.63–1.74)	0.773
Weight (kg)	70.00 (60.00–75.00)	70.50 (65.00–80.00)	0.090
BMI (kg/m^2^)	24.01 (21.97–25.93)	24.85 (23.14–26.90)	**0**.**009**
SBP (mmHg)	120 (106–136)	128 (112–141)	0.082
HR (bpm)	80 (71–94)	80 (73–86)	0.763
Smoking (n, %)	42 (65.6)	123 (58.6)	0.313
Drinking (n, %)	19 (29.7)	54 (25.7)	0.529
Diabetes (n, %)	13 (20.3)	45 (21.4)	0.848
Hypertension (n, %)	35 (54.7)	113(53.8)	0.902

SBP, systolic blood pressure; HR, heart rate.

Bold indicates statistical differences (*P* < 0.05).

### 3.3 Thymosin α1 level was positively correlated to EF value in AMI patients

**Figure 3:**
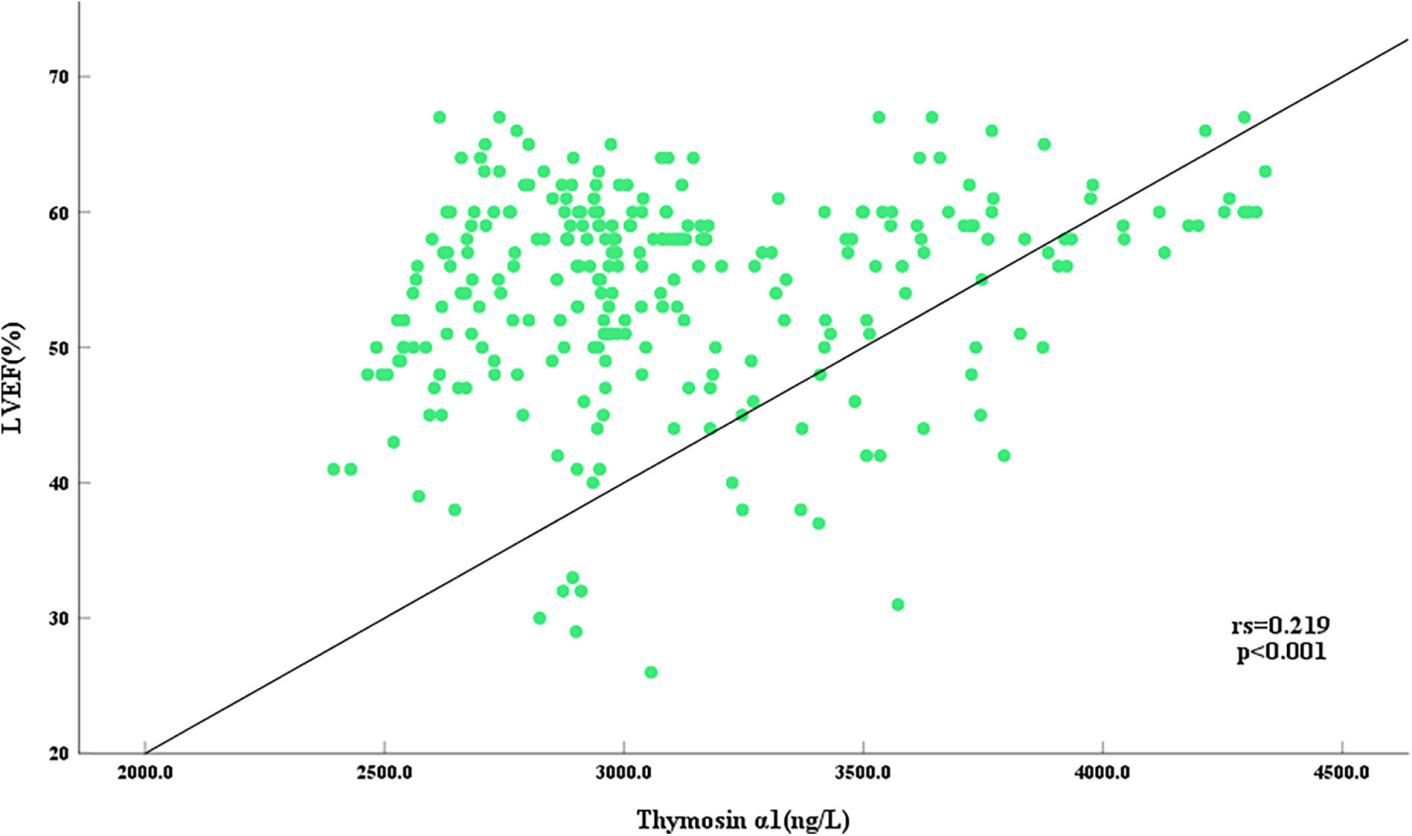
Scatter plot of correlation between serum thymosin α1 level and LVEF in AMI patients.

To further elucidate the relationship between Thymosin α1 level and cardiac function following AMI, a bivariate Spearman correlation analysis was conducted to assess the association between Thymosin α1 level and EF value in all AMI patients. The results revealed that Thymosin α1 level was positively correlated with EF value in patients post-AMI, with higher Thymosin α1 level observed as EF value increased (r = 0.219, *P* < 0.001) (Figure 3).

### 3.4 Risk factors for EF value in AMI patients

To further investigate the risk factors of cardiac function in AMI patients after opening IRA, we divided AMI patients into two groups: the EF < 50% group and the EF ≥ 50% group, with the specific number of samples in each group remaining the same as before. Firstly, a univariate comparison between the two groups of AMI patients in terms of blood routine, biochemistry, and coagulation was conducted. The results showed that the levels of white blood cell count, neutrophil cell count, and fasting blood glucose level in the EF < 50% group were significantly higher than those in the EF ≥ 50% group. There were no statistically significant differences in other blood routine indicators, biochemical parameters such as liver and kidney function, blood lipid levels, and coagulation markers between the two groups (Table 3). Secondly, we collected myocardial injury markers of the two groups and compared them. The results indicated that the expression levels of troponin I, NT-proBNP, lactate dehydrogenase, creatine kinase, and creatine kinase isoenzyme in the group with poorer cardiac function post-AMI (EF < 50%) were dramatically higher than those in the group with better cardiac function following AMI (EF ≥ 50%) (Table 4).

**Table 3:** Comparison of serum biochemical indexes among different ejection fraction groups in AMI patients.

Variable	LVEF < 50% (*n* = 64)	LVEF ≥ 50% (*n* = 210)	*P*-value
Hemoglobin (g/L)	141.0 (132.3–155.0)	146.0 (134.5–157.0)	0.190
Red blood cell (×10^12^/L)	4.64 (4.24–5.05)	4.71 (4.40–5.09)	0.414
Platelet (×10^9^/L)	214.5 (182.8–256.0)	222.5 (187.0–262.3)	0.492
White blood cell (×10^9^/L)	10.15 (8.65–12.68)	9.25 (7.68–11.03)	**0**.**013**
Neutrophil (%)	75.40 (61.50–83.60)	73.40 (62.63–81.13)	0.515
Lymphocyte (%)	18.40 (10.65–29.40)	18.15 (12.08–29.33)	0.530
Monocyte (%)	5.7 (4.3–7.2)	5.7 (4.5–7.2)	0.727
Neutrophil (×10^9^/L)	7.7 (5.9–9.5)	6.5 (4.7–8.6)	**0**.**019**
Lymphocyte (×10^9^/L)	1.6 (1.2–2.7)	1.8 (1.2–2.4)	0.870
Monocyte (×10^9^/L)	0.6 (0.4–0.7)	0.5 (0.4–0.7)	0.131
Kalium (mmol/L)	3.7 (3.5–4.0)	3.8 (3.4–4.0)	0.696
Natrium (mmol/L)	138.0 (136.0–140.8)	139.0 (136.0–140.0)	0.818
Chlorinum (mmol/L)	103 (100–105)	102 (100–104)	0.468
Glucose (mmol/L)	7.06 (6.13–8.58)	6.45 (5.52–8.30)	**0**.**001**
Glycated hemoglobin (%)	6.0 (5.7–6.5)	6.0 (5.7–6.6)	0.670
Total Protein (g/L)	72 (68–77)	72 (69–76)	0.467
Albumin (g/L)	42.0 (40.0–43.3)	42.0 (40.0–44.0)	0.664
Alanine aminotransferase (U/L)	35.5 (24.3–54.5)	36.5 (27.0–46.3)	0.765
Creatinine (umol/L)	75.5 (60.0–94.8)	71.0 (62.0–81.0)	0.141
Urea nitrogen (mmol/L)	5.9 (4.9–7.8)	5.5 (4.8–6.7)	0.062
Uric acid (*μ*mol/L)	373.0 (295.3–427.5)	353.0 (285.8–418.0)	0.107
Triglyceride (mmol/L)	1.085 (0.735–1.923)	1.380 (0.880–1.920)	0.095
Total cholesterol (mmol/L)	4.860 (4.093–5.605)	4.970 (4.280–5.720)	0.300
High-density lipoprotein (mmol/L)	1.07 (0.83–1.28)	1.02 (0.85–1.21)	0.463
Low-density lipoprotein (mmol/L)	3.09 (2.55–3.60)	3.18 (2.49–3.77)	0.180
Apolipoprotein A1 (g/L)	1.15 (0.97–1.28)	1.15 (1.04–1.28)	0.712
Apolipoprotein B (g/L)	0.91 (0.72–1.01)	0.95 (0.80–1.10)	0.106
Prothrombin time (s)	11.7 (10.5–11.9)	11.1 (10.5–11.9)	0.081
Apolipoprotein E (g/L)	3.76 (3.24–4.80)	3.76 (3.04–4.71)	0.710
Lipoprotein α (g/L)	23.35 (14.03–36.23)	16.40 (9.40–29.90)	0.077
International normalized ratio	1.02 (0.96–1.05)	0.96 (0.91–1.04)	0.081
Activated partial thromboplastin (s)	26.8 (23.9–29.6)	26.0 (22.9–28.5)	0.253
Thrombin time (s)	16.5 (15.9–17.4)	16.5 (15.8–17.2)	0.729
Fibrinogen (g/L)	2.699 (2.388–3.163)	2.636 (2.356–2.992)	0.172
D-dimer (mg/L)	0.39 (0.22–0.81)	0.32 (0.20–0.54)	0.095
C-reactive protein (g/L)	3.345(0.499–8.628)	1.110(0.499–6.820)	0.349

Bold indicates statistical differences (*P* < 0.05).

**Table 4:** Comparison of myocardial injury markers and serum thymosin α1 levels among different ejection fraction groups in AMI patients.

Variable	LVEF < 50% (*n* = 64)	LVEF ≥ 50% (*n* = 210)	*P*-value
Troponin I (μg/L)	158.86 (44.18–253.73)	46.67 (12.64–105.35)	**<0**.**001**
N-terminal pro-B-type natriuretic peptide (pg/ml)	2,943 (1,083–5,919)	972 (556–1,957)	**<0**.**001**
Aspartic aminotransferase (U/L)	129 (40–282)	92 (46–205)	0.155
Lactate dehydrogenase (U/L)	1,083 (679–1,858)	561 (373–810)	**<0**.**001**
Creatine kinase (U/L)	3,563 (1,668–5,353)	1,474 (630–2,717)	**<0**.**001**
Creatine kinase isoenzyme (U/L)	286.75 (133.50–413.13)	140.95 (63.75–239.08)	**<0**.**001**
Thymosin α1(ng/L)	2,913.28 (2,648.35–3,247.05)	3,002.50 (2,846.48–3,497.68)	**0**.**006**

Bold indicates statistical differences (*P* < 0.05).

### 3.5 OR for AMI-related EF value using logistic regression analysis

Logistic regression analysis was conducted on all parameters including thymosin α1 that were notably different by univariate analysis between the EF < 50% group and the EF ≥ 50% group. Risk factors with a remarkable difference in AMI-related EF value included Thymosin α1 (OR = 1.000808, *P* = 0.048), NT-proBNP (OR = 0.999844, *P* < 0.001), and creatine kinase (OR = 0.999638, *P* < 0.001) (Table 5).

**Table 5:** Multivariate logistic regression analysis of LVEF ≥ 50% after AMI.

Variable	Β value	Wald *χ*^2^	OR（95% CI）	*P*-value
Thymosin α1 (ng/L)	0.00081	3.899	1.000808（1.000006–1.001611）	**0**.**048**
N-terminal pro-B-type natriuretic peptide (pg/ml)	−0.00016	13.250	0.999844（0.999760–0.999928）	**<0**.**001**
Creatine kinase (U/L)	−0.00036	25.209	0.999638（0.999496–0.999779）	**<0**.**001**

Bold indicates statistical differences (*P* < 0.05).

### 3.6 The ROC analysis of thymosin α1 for predicting AMI-induced cardiac dysfunction

**Figure 4:**
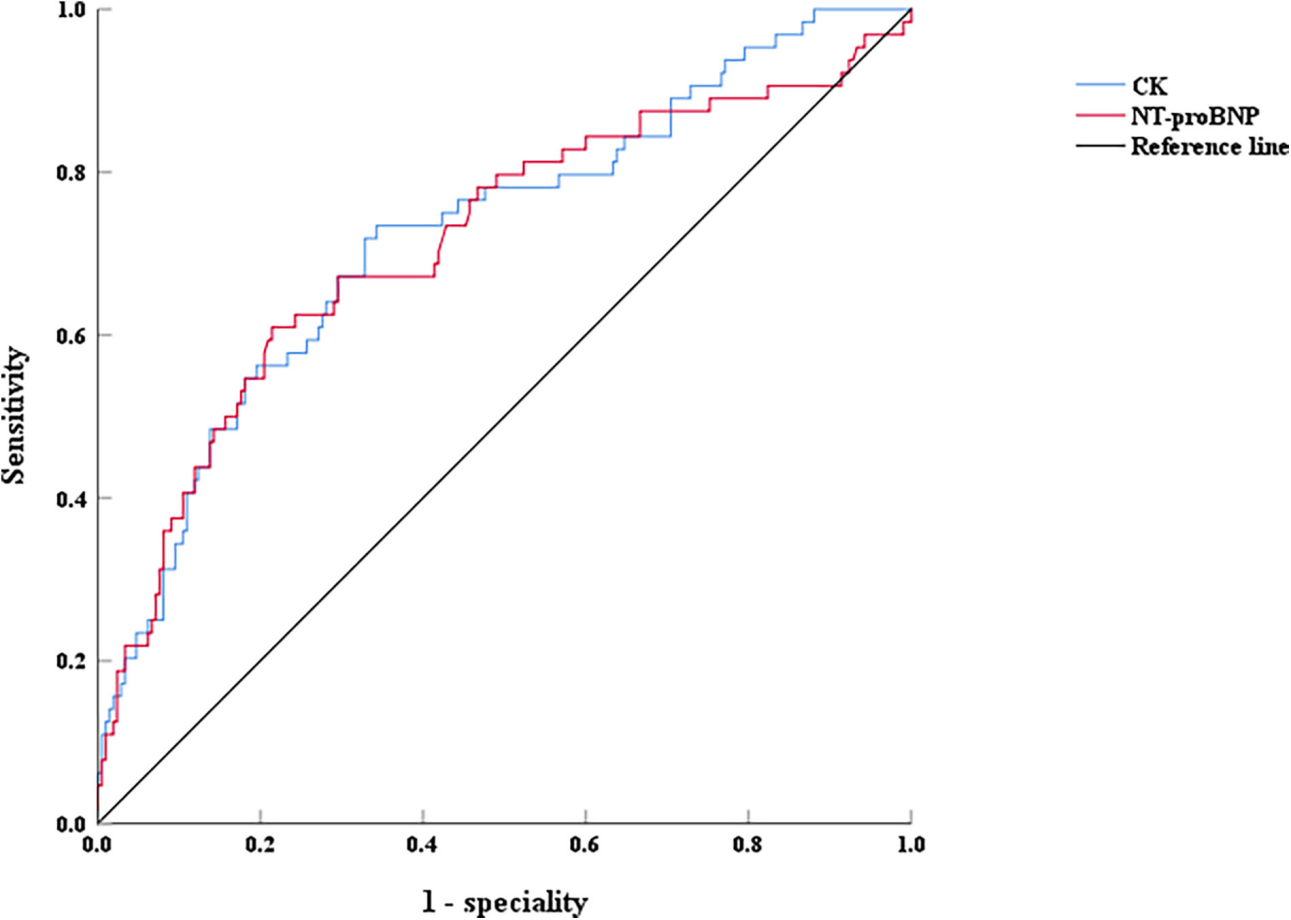
The ROC curves of NT-proBNP and CK on LVEF < 50% after interventional treatment in AMI patients, CK: creatine kinase.

**Figure 5:**
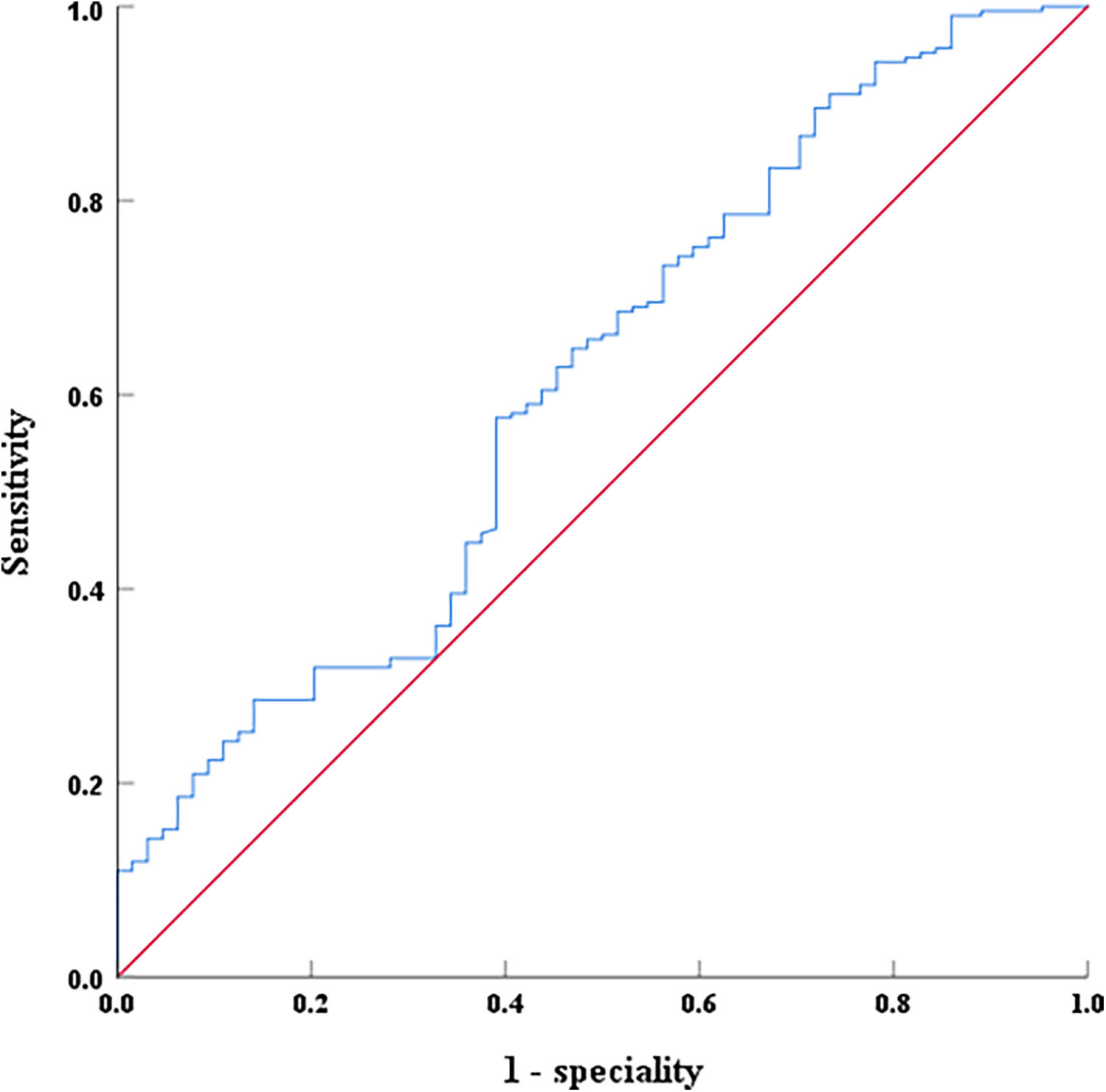
The ROC curves of serum thymosin α1 levels on LVEF ≥ 50% after interventional treatment in AMI patients.

Thymosin α1, NT-proBNP, and creatine kinase were analyzed using the ROC curve to evaluate their predictive value for EF decline caused by AMI. For predicting LVEF < 50% post-AMI, the area under the curve (AUC) of NT-proBNP was 0.714 (0.635–0.792, *P* < 0.001), with an optimal cutoff of 2,163 pg/ml, and the sensitivity and specificity were 60.9% and 73.4%, respectively (Figure 4). The AUC of creatine kinase was 0.724 (0.651–0.798, *P* < 0.001), with an optimal cutoff of 1,943 pg/ml, and the sensitivity and specificity were 78.6% and 65.7%, respectively (Figure 4). However, for predicting LVEF ≥ 50% in AMI patients during hospitalization, the AUC of Thymosin α1 level was 0.614 (0.533–0.694, *P* < 0.001), with an optimal cutoff of 2,961.72 ng/L, and the sensitivity and specificity were 57.6% and 60.9%, respectively (Figure 5).

## 4 Discussion

This study primarily revealed a positive correlation between thymosin α1 level and cardiac function following AMI. Specifically, higher expression levels of thymosin α1 were associated with improved cardiac function after AMI. Additionally, the ROC curve analysis indicated that thymosin α1 was an independent predictor of cardiac function (EF ≥ 50%) following AMI, with its sensitivity and specificity being 57.6% and 60.9%, respectively. Our team formerly reported the relationship between thymosin α1 and cardiac function after acute anterior ST-segment elevation myocardial infarction in the Chinese Journal of Interventional Radiology. Building upon this prior research, an expanded sample size and a broader range of AMI types were examined to draw more reliable conclusions. As far as we are aware, this study was the first to focus on thymosin α1 as a predictive marker for cardiac function following AMI, potentially offering a novel target for the early clinical detection and treatment of HF post-AMI.

A comparison was initially conducted between the control group and the AMI group, followed by a subgroup analysis based on whether the EF value from echocardiography during hospitalization post-AMI exceeded 50%. Myocardial ischemia, hypoxia, and even necrosis occur after AMI, leading to myocardial damage (19). Reperfusion injury of the myocardium after culprit vessel recanalization can further exacerbate myocardial damage (19). Therefore, during the early hospitalization period of AMI, some patients may begin to experience a decline in cardiac function, and the occurrence of HF is also possible (19). Clinically, the EF value from early echocardiography can be used to assess myocardial contractility and indirectly reflect cardiac function post-AMI. A lower EF value indicates worse cardiac function and a higher long-term risk of HF for AMI patients, which in turn suggests a poorer long-term prognosis (20, 21). Therefore, the subgroup analysis in this study based on the EF value during hospitalization post-AMI was justified. Our results indicate that in addition to thymosin α1, the traditional HF marker NT-proBNP and the myocardial injury marker creatine kinase were retained in the logistic multivariate analysis. NT-proBNP is a well-recognized marker of HF, with higher NT-proBNP values correlating with more severe HF. Previous studies have shown that BNP is associated with the larger size of myocardial infarction and the reduction of cardiac function, and is also related to the long-term adverse prognosis of AMI patients (22, 23). Our study concluded that there was a negative correlation between the expression level of NT-proBNP post-AMI and the EF value which was consistent with previous research. Furthermore, our study also identified the predictive value of creatine kinase for the reduction of cardiac function post-AMI. Creatine kinase is a traditional marker of myocardial damage area following AMI and is typically associated with the extent of myocardial damage post-AMI (24, 25), which explains its predictive value for the reduction of cardiac function post-AMI. The retention of these traditional predictive markers of myocardial injury post-AMI for cardiac function in this study further underscores the reliability of the study's conclusions.

Thymosin α1 is a bioactive peptide containing 28 amino acid residues, obtained by cleavage of prothymosin α (composed of 109 amino acid residues) with asparagine endopeptidase (26). It may be involved in cell cycle regulation and indirectly affect transcription and/or DNA replication processes (27). Thymosin α1 can bind to receptors on or near the membrane, triggering biological signal cascade reactions and participating in various physiological processes in the body, especially the regulation of immune responses (28). Currently, the inflammatory response plays a crucial role in the progression of AMI, which can lead to an increased infarct size and reduced cardiac function. Particularly, monocytes and macrophages significantly contribute to the infarct size and remodeling after AMI. Previous studies have demonstrated that anti-inflammatory macrophages (M2-type macrophages) or cardiac repair macrophages *in situ* post-AMI can alleviate post-infarction myocardial inflammation by secreting anti-inflammatory cytokines, thereby reducing ventricular remodeling and ultimately improving cardiac function (29, 30). A study has shown that thymosin α1 can improve the efficacy of chemotherapy for breast cancer by reversing M2 polarization of efferocytosis-activated macrophages (31). In addition, the latest study indicated that both the exogenously provided and the adenovirus-produced thymosin α1 mediate the tumor-associated macrophages M2 polarization via CD8+ T cells, thereby enhancing the anti-tumor effect of adenovirus (32). Therefore, we could infer that the high expression of thymosin α1 post-AMI may promote the transformation of macrophages infiltrating the myocardium into anti-inflammatory (M2 type) macrophages, thereby exerting a cardioprotective effect and improving cardiac function. This may explain the high expression of thymosin α1 observed in the group with better cardiac function following AMI in our study.

In addition to the association between monocytes/macrophages and cardiac function in AMI, recent studies have demonstrated that regulatory T cells (Tregs), acting as immune response modulators, can also exert cardioprotective effects after AMI and improve cardiac function by modulating the polarization of macrophages (33, 34). Previous studies have shown that in the treatment of cytomegalovirus infection, thymosin α1 could enhance the function of regulatory T cells (Tregs) and reduce Treg cell senescence (35). Furthermore, thymosin α1 could also promote the development and tolerance of Treg cells by regulating DC cells (36). In conclusion, it could be speculated that the up-regulation of thymosin α1 following AMI might reduce the myocardial inflammatory response by enhancing the function of Treg, thereby exerting a cardioprotective effect and improving cardiac function. However, the specific mechanisms underlying these effects require further investigation. To our knowledge, this was the first report demonstrating that thymosin α1 expression was increased following AMI, with higher expression levels correlating with better cardiac function. Through this study, we have uncovered for the first time that thymosin α1 might serve as a protective factor against myocardial injury post-AMI. This finding could provide a novel target for clinically improving cardiac function following AMI and a new therapeutic strategy for reducing the incidence of HF post-AMI.

Although the sample size was increased compared with our previous study, it remained relatively small. The relatively small sample size of the control group and the larger sample size of the case group in our study may influence subsequent research outcomes, such as the moderate sensitivity and specificity of thymosin α1 (AUC = 0.614). Moreover, our study exclusively focuses on Chinese individuals. We plan to increase the total sample size, including additional control group samples and more diverse populations, to a enable more comprehensive analysis. Secondly, the blood samples collected in this study were taken during patients' hospitalization. As myocardial infarction is a dynamic process, we will continue monitoring thymosin α1 levels after coronary artery revascularization to further validate our research conclusions. Thirdly, we identified elevated thymosin α1 as a novel correlate of post-AMI cardiac function recovery. While its predictive power for thymosin α1 remains moderate (AUC = 0.614), this emerging biomarker warrants further investigation. Importantly, our data confirm the expected inverse relationships between LVEF and traditional markers, NT-proBNP (AUC = 0.714) and CK (AUC = 0.724), demonstrating methodological validity while contextualizing thymosin α1's comparatively modest effect size. Subsequent investigations will incorporate serial thymosin α1 measurements at standardized pre-intervention and post-intervention timepoints to delineate its prognostic utility. And the specific protective mechanisms and underlying signaling pathways remain to be elucidated. Lastly, this was a retrospective study which resulted in deficiencies in the data collection process and possible selection bias. Future prospective multicenter studies should investigate thymosin α1 as a potential cardioprotective agent following AMI and elucidate its role in reducing HF incidence.

## 5 Conclusions

In conclusion, this study discovered that in comparison with the control group, the expression level of thymosin α1 in peripheral blood after AMI was notably elevated, and the better the cardiac function, the higher the expression level of thymosin α1. Thymosin α1 might be a biological indicator for the enhancement of cardiac function following AMI, and it could also be an important bioactive substance for the treatment of cardiac dysfunction after AMI. A prospective, randomized, and multicenter study should be devised to further validate the efficacy of thymosin α1 in improving cardiac function after AMI. Additionally, the two traditional biomarkers for cardiac function post-MI were still maintained in this study, indicating that these traditional predictive markers should not be disregarded.

## Data Availability

The raw data supporting the conclusions of this article will be made available by the authors, without undue reservation.
